# MULTIDISCIPLINARY AND MOTIVATIONAL INTERVENTION FOR THE TREATMENT OF LOW INCOME BRAZILIAN OBESE ADOLESCENTS: PILOT STUDY

**DOI:** 10.1590/1984-0462/;2018;36;2;00014

**Published:** 2018-04-23

**Authors:** Andrea Rocha Filgueiras, Ana Lydia Sawaya

**Affiliations:** a Universidade Federal de São Paulo, São Paulo, SP, Brasil.

**Keywords:** Dietary behavior, Obesity, Motivation, Body composition, Comportamento alimentar, Obesidade, Motivação, Composição corporal

## Abstract

**Objective:**

To test a multidisciplinary and motivational intervention for the treatment of Brazilian obese and low-income adolescents (Z score>2 BMI-for-age) that used nutritional counseling without dietary control.

**Methods:**

An intervention protocol was developed including periodical nutritional education workshops, individual nutritional counseling guided by the stages of eating behavior of the Trans Theoretical Model of Behavior Change, physical exercise, psychological counseling, recreational activities, and clinical follow-up for 13 months in a sample of 21 adolescents (11-17 years old).

**Results:**

The rate of treatment withdrawal (9.5%) was lower than that seen in dietary control studies (30-60%). Initially, 70% of the sample was in the pre-contemplation behavior stage and, in the end, 100% of the remaining adolescents were in the stages of action or maintenance. There was a mean reduction in BMI-for-age (p=0.038) and visceral fat (M±SD=3.67±1.19 and 2.78±0.78 cm, p=0.02, initial and final, respectively). The percentage of fat mass decreased and that of lean mass increased (42±5 and 38±8, p=0.04, 58±6 and 61±8%, p=0.03, respectively).

**Conclusions:**

The intervention seems to be effective in generating a lifestyle change, accompanied by anthropometric profile and body composition improvement. The intervention protocol may offer easy adaptation and low-cost methodology for health services, with high adherence and low abandonment rates.

## RESUMO

**Objetivo:**

Testar uma intervenção multidisciplinar e motivacional para o tratamento de adolescentes obesos brasileiros e de baixa renda (escore Z>2 IMC-para-idade) que utilizou o aconselhamento nutricional sem controle dietético.

**Métodos:**

Desenvolveu-se um protocolo de intervenção que incluiu a realização periódica de oficinas de educação nutricional, aconselhamento nutricional individual com auxílio do modelo transteórico de mudança do comportamento, prática de exercícios físicos, aconselhamento psicológico, atividades recreativas e acompanhamento clínico, durante 13 meses, em uma amostra de 21 adolescentes (11-17 anos).

**Resultados:**

A taxa de desistência do tratamento (9,5%) foi menor do que a verificada em estudos de controle dietético (30-60%). No início, 70% da amostra se encontrava no estágio de comportamento denominado pré-contemplação (sem intenção de mudança) e, ao final, 100% dos adolescentes que permaneceram passaram aos estágios de ação ou manutenção (mudaram o comportamento ou mantiveram a mudança por mais de seis meses). Observou-se diminuição média de IMC-para-idade (15%, p=0,038) e de gordura visceral (gordura inicial de 3,67±1,19 e final de 2,78±0,78 cm, p=0,02). O percentual de massa gorda diminuiu e o de massa magra aumentou, quando comparados os valores médios iniciais e finais (42±5% e 38±8%, p=0,04; 58±6% e 61±8%, p=0,03, respectivamente).

**Conclusões:**

A intervenção parece eficaz para gerar mudança de estilo de vida, acompanhada de melhoria do perfil antropométrico e de composição corporal. O protocolo de intervenção pode oferecer metodologia de fácil adaptação e baixo custo para serviços de saúde, com alta adesão e baixa taxa de abandono.

## INTRODUCTION

The overweight and obesity epidemic in adolescence is a major cause for concern in the world.^1^ ^,^ ^2^ The dietary environment has been described as unhealthy and obesogenic, composed of high energy density foods, at low cost, poor in nutrients, but widely available, and object of intense advertising in different medias.^3^

This environment increases the incidence of dietary behavior disorders, promotes physical inactivity, irregular sleep pattern and excessive screen time,^4^ leading to increasing morbimortality.^5^ It is extremely important to intervene in the prevention of this epidemic;^6^ however, efforts should be made to treat the Young people who already present with excess weight.^7^

Therefore, it is necessary to have changes in lifestyle, reducing sedentary habits and increasing energy expenditure, by including daily physical exercises and reducing energy intake.^8^ The adoption of new habits, however, requires a multidisciplinary and motivational approach, that is, it is not enough to simply impose these two practices.^9^ ^,^ ^10^ ^,^ ^11^ Studies indicate that the individuals with adequate motivation to change habits can reach their goal more easily than those without satisfactory motivation.^12^

The objective of this intervention was to test an innovative protocol to treat low-income Brazilian obese adolescents, for a 13-month period, which included motivation for change, nutritional educational, promotion of physical activities, clinical follow-up and psychological counselling, as well as recreational and integration activities without control or restriction of the intake of foods and beverages.

## METHOD

This is an initial study that proposes a multidisciplinary and motivational intervention in obese adolescents aged from 11 to 17 years, of both genders (Z score>2 of BMI-for-age),^13^ living in low-income neighborhoods in the city of São Paulo.

**Figure 1:**
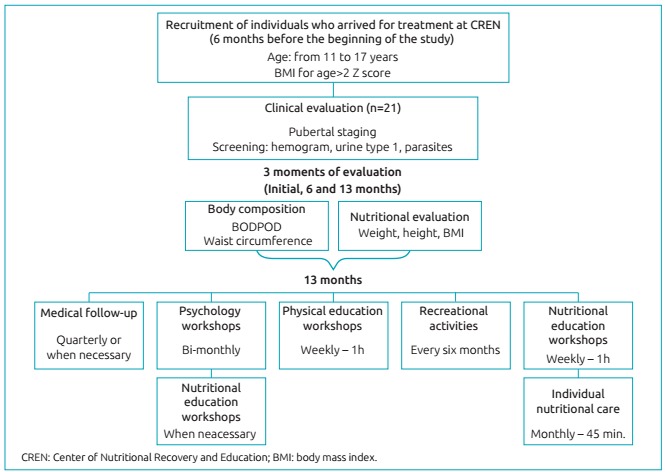
Study design.

The sample included individuals who arrived at the Center of Nutritional Recovery and Education (CREN)^14^ looking for nutritional treatment, referred by primary care units or coming from spontaneous demand in the six months before the beginning of the protocol (Figure 1). Twenty-one adolescents accepted to participate and signed, together with their tutors, after explanations, the informed consent form approved by the Research Ethics Committee of Universidade Federal de São Paulo (Unifesp - CEP n. 125 855/12). The exclusion criteria were: twins, adolescents who had congenital conditions, genetic syndromes, hormone disorders that affect growth, those with previous use of androgenic anabolic steroids or psychotropic, and teenage pregnant girls.

The participants were submitted to screening to verify the general health status, which included an ova and parasite test, urine test type hemogram, TSH dosage, and I free T_4_ and free T_3_. The positive cases for anemia or infection were treated and, afterwards, included in the protocol; the ones presenting any other change were excluded. This procedure aimed at preventing infections, parasites or anemia to affect the nutritional evolution of adolescents during intervention.

All participants went through individual nutritional care in a CREN office (Figure 1), with the objective of helping them overcome the difficulties and barriers involved in changing dietary habits, reinforcing the positive aspects of the changes that had already been made. Nutritional intervention was not based on controlling the intake of foods or energy, nor on the results obtained by individual dietary surveys, but instead, on the use of dietary behavioral change stages obtained by the algorithm of the Transtheoretical Model (TTM), to address the motivation and the elaboration of goals towards dietary behavioral changes.^15^ During nutritional counselling, three goals were established (easy, medium and difficult) to be followed-up and changed along the treatment. The algorithm was applied in three moments: the first appointment, after six months and at the end of the 13 months. This technique maps five stages of promptness for behavioral change: pre-contemplation, contemplation, preparation, action and maintenance.

 The pre-contemplation stage is characterized by the lack of will to change, resistance to recognize the problem and change it, or total unawareness of the need for change.^11^ Therefore, for the ones who were in the pre-contemplation stage, the focus of counselling was to help them understand and accept the importance of changing consumption patterns, increasing their knowledge about healthy dietary habits and becoming more aware of their dietary practices.

The contemplation stage identifies individuals who are aware of their problem and seriously consider changing their behavior, but have not yet taken any initiative, do not know where to start or do not feel ready.^15^ The service addressed to adolescents in this stage aimed at reinforcing and facilitating the process of change, increasing their confidence in the ability to adopt the nutritional recommendations.

The stage of preparation is characterized by the fact that the individual wants a change in behavior in the near future, claiming to be motivated and seeing that change could happen in the following months. For adolescents in this stage, there were goals to assist their wish for change, by defining a plan of action addressed to the modification of dietary habits.

For those in the action stage who had already begun to change their behavior, the motivation was reinforced by emphasizing the advantages of adopting a healthier lifestyle. In this stage, strategies to continue to change were established to make sure that the changes in dietary habits would be maintained.

The maintenance stage is characterized by the fact that the change may have occurred more than six months earlier. Adolescents were then encouraged to maintain the changes and habits acquired by reinforcement and memories of the path they took to reach the maintenance stage. Finally, they were stimulated to develop the necessary skills to face new difficulties.

An algorithm was adopted for the changes in general dietary behavior, composed of four questions,^16^ and not the complete questionnaire with 38 questions,^17^ for being simpler for practice in health services. Besides, the monitoring of stages in nutritional care was a supporting information of the motivation for change, and not the only strategy for treatment. The evaluation of the stages in which most adolescents was in helped the preparation of the content of the workshops.

The nutritional education workshops were conducted in CREN (Figure 1), with the following contents: fruits of the time; obesity and metabolism; self-knowledge and self-image; perception of hunger and satiety; planning and goals; ten steps towards healthy diets; eating during parties and holidays; industrialized foods; and practice of physical exercises.

The physical education workshops (Figure 1) were conducted in CREN and in a park. In most interventions, the physical activities were conducted together with the nutritional education workshops using games, tournaments etc. The adolescents were stimulated to increase their daily activities, such as walking to school or riding a bike to CREN. In these occasions, they learned exercises to practice at home or in parks. Every week, one adolescent was selected to take home a mini trampoline.

The Psychology workshops in CREN took place, in average, once every two months, and focused on the integration and on the socialization between participants, with the following subjects: understanding of the body signals, anxiety and self-control, how to deal with setbacks, knowing the body and its shape, dichotomous thinking, breathing and relaxation techniques. Individual psychological care was carried out according to the patients’ need.

The workshops were organized at two different times (morning and afternoon), according to the students’ workload, and were conducted in an alternate shift in relation to school. The workshops lasted from 2 to 3 hours and included 10 to 11 students. There was no distinction of sex, pubertal stage, and age between the participating groups.

Three recreational activities were carried out (Figure 1) with the objective of increasing the connection between the research team and the participants. During the tours, there were nutritional education workshops according to the corresponding cycle and to the physical activity, using the local space. Relatives or tutors could also take part in the weekly activities and in the tours.

Medical follow-up (Figure 1) was conducted by a single professional at first, after 6 and 13 months of hospitalization or according to the need. During the appointments, the pubertal stage was assessed. Girls with breasts and pubic hair and boys with stage III genitalia were considered as pubescent.^18^

**Figure 2:**
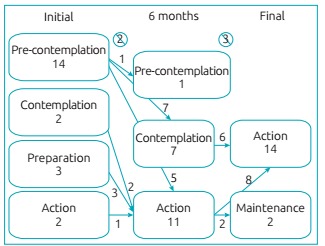
Evolution of dietary behavior stages during the intervention. Chi-square p=0.05. Description of the behavioral stages of adolescents during the intervention period. In the beginning, 14 adolescents were in pre-contemplation; 2 were in contemplation; 3 were in preparation; and 2 were in action. Two of them changed address (one in the pre-contemplation and another in the action stage). After six months of intervention, 1 adolescent continued in pre-contemplation, 7 moved to contemplation, and 11 to action. Two gave up the treatment and one left the study due to change in address. At the end, after 13 months of intervention, 16 participants concluded the protocol, of which 14 were in action, and 2, in maintenance.

The qualitative evaluation used the stages of change (Figure 2), and the quantitative evaluation used the changes in anthropometric profile and body composition (Table 1). Body composition was measured by plethysmography (BOD POD^®^ *Life Measurement Instruments ­*­- Concord, CA, EUA), and visceral and subcutaneous fat, by upper abdomen ultrasound.^19^ Waist circumference was measured according to the recommendations of the World Health Organization (WHO).^20^ For the analysis of nutritional status, the software AnthroPlus-2007, V.1.0.4 was used (WHO, Geneve, Switzerland - avaiable at http://www.who.int/growthref/tools/en/), which provides the Z score for the body mass index (BMI) and the Z score of height-for-age.

**Table 1:** Anthropometry and body composition throughout the 13 months of intervention.

	Moment of the evaluation			
	Beginning (n=21) Mean (SD)	6 months (n=18) Mean (SD)	13 months (n=16) Mean (SD)	p-value^**a**^
Visceral fat (cm)	3.67 (1.19) 0.84-6.66	3.28 (1.25) 1.12-6.33	2.78 (0.78)^#^ 1.07-4.36	0.026
Subcuta-neous fat (cm)	2.72 (1.0) 1.54-5.25	2.67 (0.83) 1.13-4.09	2.63 (1.09) 1.09-4.49	0.527
Fat mass (%)	42 (5) 33.3-52.2	42 (7) 29.2-52.9	38 (8)* 26.3-52.3	0.043
Lean body mass (%)	58 (6) 47.8-68.8	58 (7) 47.1-70.8	61 (8)* 47.7-76.7	0.032
Abdomen (cm)	103 (11.1) 85.3-136.0	102 (11.2) 88.0-135.2	99 (11.2) 76.7-12.3	0.099
Zscore BMI	2.70 (0.51) 12.00-4.07	2.55 (0.50) 1.72-3.89	2.30 (0.73)* 0.71-3.75	0.038

SD: standard deviation; ^#^p<0.05 when compared to the beginning of the treatment; *p<0.05 when compared to six months of treatment; ^a^ANOVA for measurements repeted in three momtns, adjusted for multiple comparisons: Bonferroni; BMI, body mass index.

The statistical analyses were conducted using the software *Statistical Package for the Social Sciences* (SPSS), version 20 (Chicago, IL, USA), and the statistical significance was established at p*<*0.05. For the analysis of the flow between dietary behavior stages, the chi-square test was used. Anthropometric and body composition changes were calculated by the analysis of variance (ANOVA), adjusted for multiple comparisons using the Bonferroni test.

## RESULTS

This is a pilot study, with a convenience sample composed of 12 girls and 9 boys, mean age of 14±1.5 years, of which 90% were pubescent. Throughout intervention, three adolescents abandoned the treatment due to changes of address or city, and two gave up. Therefore, after excluding the ones who abandoned treatment due to change of address, the desistance rate was 9.5%. In general, the total loss of the study was 23.8% of the initial sample. The mean per capita monthly income was R$ 247.32±126.44, and four participants reported not having a stable income in the family.

In the beginning of intervention, approximately 70% of the participants were in the pre-contemplation stage; after six months, 60% of them changed to the action stage (Figure 2). All participants who stayed until the end of the study reached the stages of action or maintenance.

The evolution of the anthropometric profile and body composition (Table 1) had concomitant reduction in the Z score of BMI-for-age (-0.40) and visceral fat (-0.89 cm), besides gain of lean mass (3%) and reduced percentage of body fat (4%). There was no significant difference when the sample was stratified by sex.

## DISCUSSION

This pilot study aimed at presenting an innovative motivational intervention based on nutritional counselling, on the promotion of physical activity, on the clinical follow-up and psychological counselling, as well as on recreational and integration activities, without specifically controlling the intake of foods and beverages, and assisted by the stages of TTM for dietary behavioral change in obese, low income adolescents.

The first major effect of the intervention was the low desistance rate in the treatment (Figure 2). It is known that motivational processes may play a crucial role in behavioral change and in the maintenance of good practices towards a healthy life among adolescents.^21^ Many studies that included strategies of change in lifestyle and physical activity without, however, having a multidisciplinary approach or systematic motivational intervention showed desistance rates of 30 to 60% in the first six months of treatment.^22^

Recent studies report that the level of motivation includes, in a conscious or unconscious manner, any effort to change thoughts, emotions, attention, impulses and behavior, in order to reach and maintain personal goals^23^, such as controlling dietary intake or making healthy, even if less palatable choices. Studies also show that, during a slimming treatment, obese children and adolescents usually present higher sensitivity to reward and less inhibitory control in comparison to thin children and adolescents^23^ ^,^ ^24^ ^,^ ^25^. Besides, low motivation for change is predictive of the abandonment of the goals and the treatment^23^.

Another aspect that stands out in this study is the fact that the workshops and the group activities were conducted with only 10 to 11 adolescents, which should have facilitated the interaction and the collaboration between participants, as well as the long-term adherence to the protocol. Besides, the students in the workshops attended the same school and belonged to the same community, so they were friends. According to the WHO, the participation of friends in the development and implementation of interventions for socially disadvantaged groups leads to more acceptability, skills and sense of belonging to the group.^26^

It is Worth to mention that this was a high-intensity intervention,^27^ since it included more than 156 hours of group or individual activities for 13 months. However, despite the high intensity of the intervention, this study proposes its reproducibility in health services, because the adoption of small groups lasting 2-3 hours for the treatment of chronic conditions constitutes an intervention structure used in basic care services.

The magnitude of the improvement in the nutritional profile (Z score of BMI-for-Age-0.40) was lower than that found in other studies^1^ ^,^ ^5^ ^,^ ^28^ that used dietary restriction and inclusion of vigorous physical activity at least three times a week as strategies to treat excess weight. However, recent studies have shown that the reduction in the Z score of BMI in 0.2 is already associated with clinically significant improvement in nutritional status.^29^ On the other hand, a treatment that involves dietary control and vigorous physical activity may reduce adherence and become unviable for health services. Besides, the long-term positive impact of the treatment with restrictive diets and faster weight loss has been strongly questioned.^28^ ^,^ ^30^

The main limitation of this study is the small sample size. Therefore, the current results must be considered as a pilot study. We are currently developing a broader program, extending the intervention to 18 months, with a larger population of children and adolescents with excess weight.

Finally, the methodology presented in this pilot study provides initial evidence that a motivational and multidisciplinary intervention can be efficient to generate changes in lifestyle, followed by the improved anthropometric profile and body composition. Besides, this intervention protocol may offer a low-cost methodology, easy to be adapted for health services, with low abandonment rates.
